# Innovation of Word Order Harmony Across Development

**DOI:** 10.1162/OPMI_a_00010

**Published:** 2017-09-01

**Authors:** Jennifer Culbertson, Elissa L. Newport

**Affiliations:** 1 School of Philosophy, Psychology, and Language Sciences, University of Edinburgh; 2 Department of Neurology, Georgetown University

**Keywords:** learning biases, language acquisition, artificial language learning, regularization, word order

## Abstract

The tendency for languages to use *harmonic* word order patterns—orders that place heads in a consistent position with respect to modifiers or other dependents—has been noted since the 1960s. As with many other statistical typological tendencies, there has been debate regarding whether harmony reflects properties of human cognition or forces external to it. Recent research using laboratory language learning has shown that children and adults find harmonic patterns easier to learn than nonharmonic patterns (Culbertson & Newport, 2015; Culbertson, Smolensky, & Legendre, 2012). This supports a link between learning and typological frequency: if harmonic patterns are easier to learn, while nonharmonic patterns are more likely to be targets of change, then, all things equal, harmonic patterns will be more frequent in the world’s languages. However, these previous studies relied on variation in the input as a mechanism for change in the lab; learners were exposed to variable word order, allowing them to shift the frequencies of different orders so that harmonic patterns became more frequent. Here we teach adult and child learners languages that are consistently nonharmonic, with no variation. While adults perfectly maintain these consistently nonharmonic patterns, young child learners innovate novel orders, changing nonharmonic patterns into harmonic ones.

## AN APPARENT PREFERENCE FOR HARMONIC WORD ORDER PATTERNS

One of the most well-studied typological tendencies found in language concerns the ordering of heads with respect to modifiers and other dependents. Languages tend to use *harmonic* orders, placing heads either consistently before or consistently after their dependents (Dryer, 1992; Greenberg, 1963). A simple example is the noun phrase, where the head noun can be modified by elements like adjectives and number words. Almost 80% of languages in the *World Atlas of Language Structures Online* (Dryer, 2013a, 2013b) are classified as placing the noun either before both adjectives and numerals (i.e., N-Adj, N-Num) or after both of them (i.e., Adj-N, Num-N). The nonharmonic combinations of these two phrase types are much less common (i.e., N-Adj, Num-N or Adj-N, N-Num). This preference for harmony is found across many syntactic categories; for example, there is also a strong tendency for languages with head-initial Verb-Object order to have head-initial Preposition-Noun order and vice versa (see Dryer, 1992). While a number of theoretical proposals have been made to incorporate a constraint for harmony into grammatical theory (e.g., Baker, 2001; Chomsky, 1988; Travis, 1984), the statistical nature of this typological tendency has also led to alternative explanations. For example, the harmony bias has been argued to reflect general cognitive or processing constraints (e.g., favoring shorter dependencies, or simpler grammars; Culbertson & Kirby, 2016; Hawkins, 1979), or to cognition-external factors like shared in heritance among the languages sampled (Dunn, Greenhill, Levinson, & Gray, 2011), or gram maticalization (Aristar, 1991; Whitman, 2008). Thus, while there is a tendency for harmonic patterns in a number of syntactic domains, linking this tendency to the human cognitive or linguistic system requires evidence that individual language learners or users exhibit a harmony bias.

The more general issue of understanding how common features of linguistic systems relate to human cognition is a central question in cognitive science. Recent research suggests that artificial language learning experiments can provide a way to explore this—both for very general features of language, like compositionality (e.g., Kirby, Cornish, & Smith, 2008; Kirby, Tamariz, Cornish, & Smith, 2015), and for very specific types of rules, like vowel harmony (e.g., Finley & Badecker, 2009) or differential case marking (Fedzechkina, Jaeger, & Newport, 2012). Using these methods, laboratory studies have shown that learners prefer harmonic over nonharmonic word order patterns. Culbertson, Smolensky, and Legendre (2012) trained adult learners on miniature artificial languages with simple phrases consisting of a noun and an adjective or a noun and a numeral word. Each language featured a dominant order of each modifier type, but the opposite order was used in a minority of utterances. In predominantly *harmonic* conditions, both the adjective and the numeral tended to appear in the same position with respect to the noun. For example, learners might hear N-Adj or N-Num in 70% of phrases and Adj-N or Num-N in the rest. In predominantly *nonharmonic* conditions, the adjective and numeral were in different positions with respect to the noun in 70% of phrases. For example, learners might hear N-Adj or Num-N 70% of the time, Adj-N or N-Num in the rest. When tested on their production of phrases in the language, learners in harmonic conditions tended to use the majority pattern they were trained on, while learners in nonharmonic conditions tended to shift their languages toward a harmonic one. Culbertson and Newport (2015) showed that, under similar training conditions with variable input languages, child learners 6 to 7 years of age showed this shift toward harmonic word orders even more strongly.

These studies provide the first direct evidence for a link between cognition and the frequency of harmonic patterns across the world’s languages: if harmonic patterns are readily learned and used while nonharmonic patterns are more likely to be targets of change, then, all things equal, harmonic patterns will be more frequent in languages. In other words, the tendency for languages to feature harmonic orders is potentially the consequence of a pref erence or bias favoring these patterns in individuals, compounded over generations. Importantly, however, these studies have relied on input variation as a mechanism for re vealing biases in the lab. Since the languages that learners were trained on *allowed* all pos sible orders, changes to the language could be achieved without innovating new ones. The preference for harmony was revealed by the unidirectionality of these shifts in the frequency of the varying orders. However, while some natural languages allow variation (at varying levels of systematicity) in the order of nouns with adjectives or numerals, many do not. In this article we present adult and child learners with artificial languages that are perfectly and consistently *nonharmonic* in word order—that is, that always have their adjectives in a different position with respect to head nouns than their numerals—and ask whether these learners will *innovate* new orders that make nonharmonic languages more harmonic. The results of this examination will reveal whether the tendency to favor harmony is so strong that new word order patterns may be developed during learning, and, if so, in which learners this process is likely to occur.

## LEARNING A CONSISTENT NONHARMONIC LANGUAGE

### Participants

Participants were 34 adults and 45 children, all monolingual native English speakers. Adult participants were undergraduate students at Georgetown University, compensated $10 for their participation. Child participants were twenty 4- to 5-year-olds (*M* = 4; 10) and twenty-five 6- to 7-year-olds (*M* = 7; 1), recruited from daycares and camps in the Washington, DC, area.^1^

### Stimuli

Visual and auditory stimuli used for the child participants were identical to those used in Culbertson and Newport (2015). Visual stimuli were pictures of four novel objects, shown in Table 1 along with their nonce-noun labels. Each object could appear modified by one of three properties (“blue,” “spotted,” “furry”) or three numerosities (“two,” “three,” “four”), each with a pseudo-nonce label, also shown in Table 1. Stimuli used for the adult participants were identical to those used in Culbertson et al. (2012). Visual stimuli were pictures of ten novel objects (including the four shown in Table 1 below, plus six additional), which could be appear modified by one of five properties (“blue,” “green,” “furry,” “small,” “large”) or five numerosities (“two” through “six”). All labels used for adults were fully nonce, shown in Table 2. Auditory stimuli were nonce nouns alone or with a single modifier, produced using Mac Text-to-Speech (OS 10.6, speaker “Alex,” with pitch augmented using Praat; Boersma, 2001). Stimuli were displayed on a Mac computer using PsychoPy software (Peirce, 2009).

**Table 1.** Child lexicon and novel objects; note that modifiers are pseudo-nonce words.

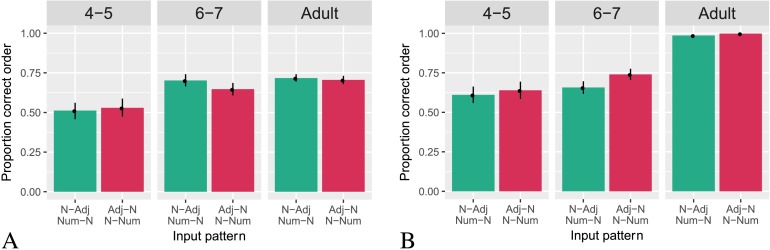	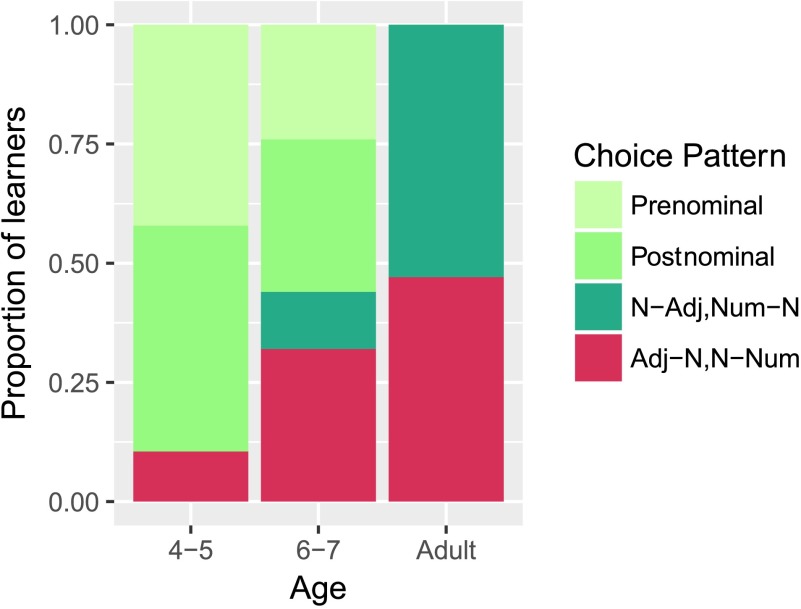	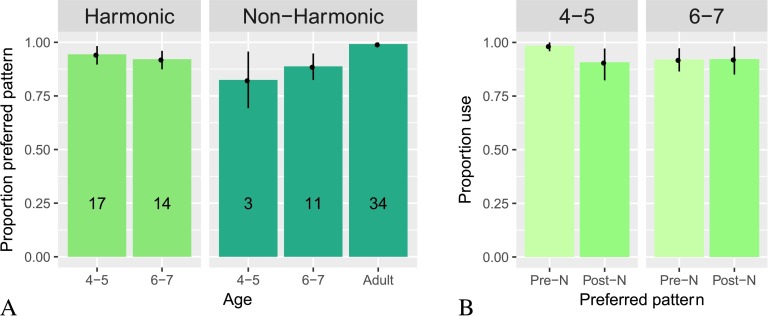	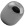	**Adjectives**	**Numerals**
				[bluθ] “blue”	[dof] “two”
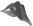					

**Table 2.** Adult lexicon.

**Noun labels**	**Adjectives**	**Numerals**
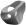		

### Manipulation

Participants in each age group were randomly assigned to one of two conditions that differed only in the word order used with each type of modifier. All languages featured phrases comprised of a noun and a single modifier—either an adjective or a numeral. Half of the participants were taught a language in which adjectives came after the noun and numerals came before (“N-Adj, Num-N”); the other half were taught a language with the opposite word orders (“Adj-N, N-Num”). These are the two *nonharmonic* languages used in Culbertson et al. (2012) and Culbertson and Newport (2015). Importantly, however, unlike the languages used in those experiments, these languages are completely and consistently nonharmonic; no phrases were included with the opposite order. We tested only nonharmonic languages in this experiment, since it is primarily these systems in which participants introduced changes in our previous studies. These patterns are of particular interest for understanding how learning biases might induce language change, thereby shaping typology.

### Procedure

Each participant was trained and tested in a single session lasting approximately 30 minutes. Sessions occurred in a quiet room with the participant seated in front of a computer. In the case of child participants, the experimenter was seated adjacent; for adults, the experimenter sat just behind the participant. Each participant was instructed that they would be learning part of a new language with the help of an “alien informant” named Glermi. The experiment progressed through a series of training phases, followed by a critical testing phase. In the first training phase, participants were introduced to the objects and their labels (20 trials total, four for each noun). Then they saw one of the objects, heard its label, and had to choose the same object from an array of all four (20 trials). They were then tested on their ability to produce the novel label corresponding to an object (20 trials). After this, they saw the object modified by either a property or a numerosity (never both) and heard a phrase describing it (48 trials, 24 noun with adjective, 24 noun with numeral). They completed a final training phase in which they heard a phrase and had to choose the corresponding picture from an array of four (48 trials, 24 adjective, 24 numeral). In the critical testing phase, they saw a picture and had to provide the corresponding phrase (48 trials, 24 adjective, 24 numeral). (For more details and examples, see Culbertson & Newport, 2015.)

## RESULTS

**Figure 1.**
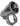
(A): Mean proportion of trials on which the correct *vocabulary* was used by participants in each age group; (B): Mean proportion of all trials on which the correct *order* was used by participants in each age group. Error bars are bootstrapped 95% confidence intervals.

Figure 1A shows mean vocabulary accuracy across trials for participants in each age group (correct vocabulary for a trial required that both noun and modifier be correct). Figure 1B shows the extent to which participants matched the input word order pattern they were exposed to.

The data were analyzed using mixed-effects logistic regression models (using the R package lme4; Bates, 2010), with all models including at least random intercepts for subject, noun, and modifier.^2^ A model predicting use of input order by age group (adult or child), condition, and their interaction revealed a significant main effect of age [χ^2^(1) = 29.52, *p* < .001], indicating increased input order use for adults compared to children. There was no significant main effect of condition [χ^2^(1) = 0.86, *p* = .35], or interaction between age and condition [χ^2^(1) = 0.04, *p* = .84]. An additional model was run comparing just the two child age groups, including age, vocabulary accuracy, condition, and their interactions as predictors. This model revealed no significant main effect of age [χ^2^(1) = 1.34, *p* = .24], vocabulary accuracy [χ^2^(1) = 2.15, *p* = .14], or condition [χ^2^(1) = 5.9, *p* = .12]. The only significant interaction was between vocabulary accuracy and age [χ^2^(1) = 4.57, *p* = .03]. The latter indicates that children in the 4- to 5-year-old group were more likely to use the input order when they also used correct vocabulary.^3^

**Figure 2.**
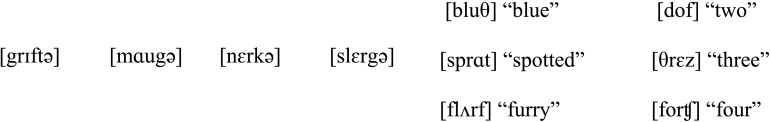
Proportion of learners in each age group who prefer each of the four possible patterns.

These results reveal that, while adults perfectly reproduce the consistent nonharmonic word order patterns they are trained on, children do not. This means that some proportion of time, children are using a different order from their input—for example, they may have consistently heard Adj-N, but produced N-Adj instead. Following Culbertson and Newport (2015), we calculated each child’s preferred pattern—that is, the pattern they used more than 50% of the time. The four possible patterns include one of the two nonharmonic patterns participants were trained on or one of the two harmonic patterns not included in the training materials: prenominal harmonic (“Adj-N, Num-N,” like English) and postnominal harmonic (“N-Adj, N-Num”). Figure 2 shows the proportion of participants in each age group who preferentially used each of these patterns, with light green and medium green indicating the harmonic patterns that were not included in the training input.

The results presented in Figure 2 show that adults are matching the pattern they were trained on. In contrast, children in the youngest age group tested, 4- to 5-year-olds, showed a preference for one of the two harmonic patterns almost across the board. The older children tested, 6- to 7-year-olds, fell between the younger children and adults, with about half preferring a harmonic pattern and half using the nonharmonic pattern on which they were trained. The difference between the two child age groups tested was confirmed by a model predict ing preferred pattern type (harmonic or nonharmonic) from age [χ^2^(1) = 4.60, *p* = .03]. As in Culbertson and Newport (2015), children show no evidence of preferring the more English-like prenominal harmonic pattern over the postnominal harmonic pattern that is unlike English.

**Figure 3.**
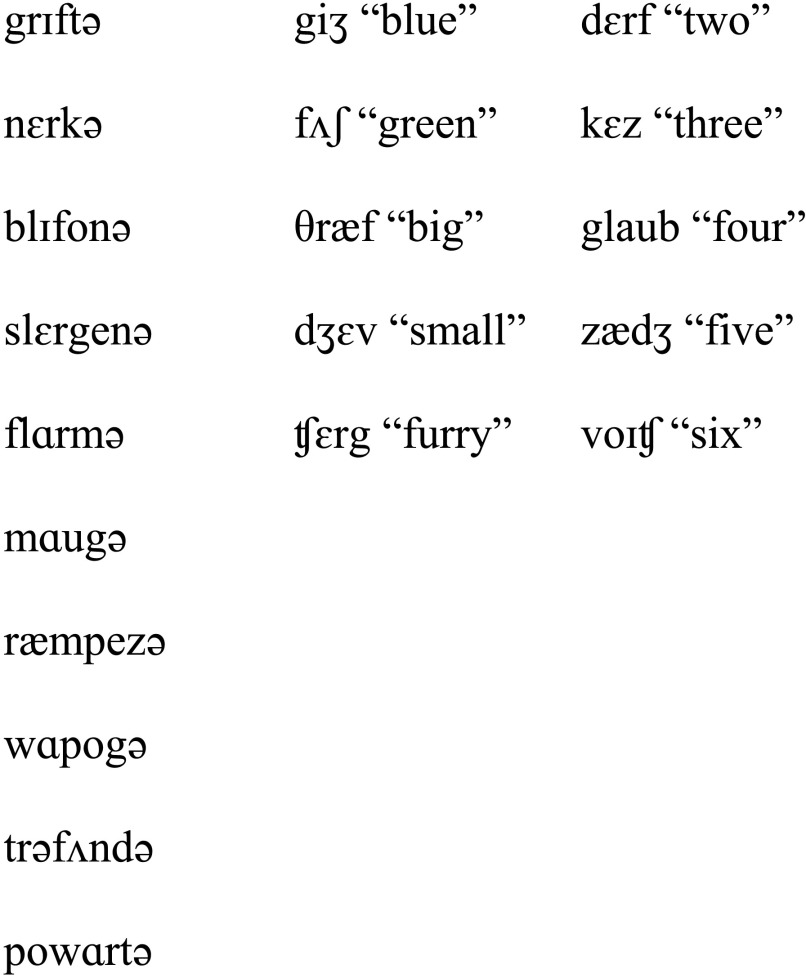
(A): Proportion use of preferred pattern for learners in each age group, depending on whether the preferred pattern was harmonic or not. This illustrates the internal consistency of word order use, given the word order that they used most frequently. Numbers on each bar indicate the number of participants included in that bar; (B): Proportion use of each specific preferred pattern for child learners in each age group, for those who preferred harmonic patterns.

Our calculation of preferred patterns indicates that a substantial proportion of child participants, particularly in the youngest age group, preferred to use a harmonic pattern rather than match their nonharmonic input. This was not a slight tendency: in almost every case, the resulting output languages were used with a high level of consistency. Crucially, even when children shifted from a nonharmonic to a harmonic pattern, they tended to use that pattern in most or all of their productions. This is shown in Figure 3. Figure 3A shows how frequently the participants used the word order that they preferred. (Note that the number of participants included in each bar varies. For example, almost all young children preferred harmonic patterns, but the few who did not still used their preferred pattern highly consistently.) Children who used harmonic patterns were slightly more consistent than those who used the input nonharmonic patterns, but this difference was not significant [χ^2^(1) = 2.84, *p* = .09; see Figure 3A]. Figure 3B illustrates the average proportion of the particular preferred pattern used, for those children who produced a *harmonic* output pattern. As the figure suggests, this high level of regularity was found for children who produced a predominantly prenominal harmonic pattern (i.e., Adj-N and Num-N) or a predominantly postnominal harmonic pattern (i.e., N-Adj and N-Num). This was confirmed by a linear regression model [proportion use of prenominal vs. postnominal harmonic pattern; χ^2^(1) = 1.68, *p* = .20]. To summarize, the degree of consistency for individual learners was similar whether or not they matched the input patterns they were trained on, and also whether or not they produced a harmonic pattern like English or unlike English.^4^

## DISCUSSION

The results of this experiment show that when trained on a consistently nonharmonic pattern of nominal word order—consistently N-Adj, Num-N *or* consistently Adj-N, N-Num—adults and children behave differently. Adults perfectly reproduced the nonharmonic pattern they were trained on, while children typically did not. Importantly, in deviating from their input, children introduced cross-category word order harmony into the language. When children harmonized the position of the noun across phrase types, they were equally likely to place the noun last (like English) or first (unlike English but common in other languages), suggesting that their behavior does not reflect straightforward transfer from English. Moreover, children used the harmonic order they innovated very consistently, in accord with Culbertson and Newport (2015), Hudson Kam and Newport (2005, 2009), and other studies where the input languages were variable (nondeterministic). Interestingly, the difference between our two child age groups suggests rapid development toward adult-like matching of nonharmonic patterns after 5 years of age.

In Culbertson et al. (2012), where adult learners were trained on variable input, adults disfavored one of the two nonharmonic patterns: Adj-N, N-Num (replicated in Culbertson, Smolensky, & Wilson, 2013). This is in fact the most typologically rare of the four patterns, suggesting the possibility of an additional bias targeting this particular nonharmonic pattern. However, as in Culbertson and Newport (2015), here we do not find any differences between the two nonharmonic conditions, either for children or for adults. In the case of adults, it seems likely that this is due to the role of variation in allowing learners to exert subtle shifts to the input without introducing new variants. By contrast, young children more readily introduce language changes, exhibiting such a strong harmony bias that differences between the nonharmonic patterns become less clear. It remains to be seen if developmental changes in knowledge of adjective or numeral syntax impact the emergence of a bias against Adj-N, N-Num when variation is present (see Culbertson & Newport, 2015, and also Goldberg, 2013).

In order to make a nonharmonic input language become harmonic, children in our experiment must innovate or, perhaps more accurately, *generalize* the ordering pattern from one phrase type to another. There are a number of possible mechanisms that may be responsible for this kind of generalization. First, in previous work we have suggested that learners are more likely to infer harmonic grammars compared to nonharmonic ones because the former are representationally simpler (Culbertson & Kirby, 2016; Culbertson & Newport, 2015). Briefly, harmonic grammars require only a single rule governing the order of nouns and modifiers, while nonharmonic grammars require additional specific rules for each modifier type. A related possibility is that, for very young learners, adjectives and numerals may not yet be distinct categories in the grammar. The latter would suggest a drive to generalize a single word order rule across lexical items rather than across categories. This may be particularly strong in children whose native language uses the same order for both of these modifier types. An important next step is therefore to investigate harmonizing behavior in children whose native language provides more robust evidence for syntactically distinguishing adjectives from numerals (Braquet & Culbertson, in press).

Taken together with previous studies, the results reported here provide evidence supporting a link between human learning and one of the most well-known typological universals of syntax. Harmonic languages are easier for both adults and children to learn, while nonharmonic patterns are more likely to be changed by learners. Children in particular dramatically shift nonharmonic patterns to harmonic ones. How do these laboratory investigations of the early stages of word order learning relate to natural language acquisition? Of course, children learning a nonharmonic language in a natural setting do *not* radically change the language they are exposed to when they produce simple phrases like a noun and an adjective. However, children presumably receive much more evidence about the syntax of the language they are acquiring before they begin producing phrases like these. Here we are requiring learners to produce phrases when they have relatively little evidence to go on, allowing us to see prior expectations or biases that we might not see in a natural language setting. Indeed, in our study, young children who were more successful at learning the lexicon of the language were less likely to shift the input word order. What our results suggest is that, even if we don’t see production errors in natural language acquisition indicating a preference for harmonic patterns—for example, in children acquiring nonharmonic languages—a strong bias may nevertheless be present. This bias continues into adulthood, but less strongly.

While the cognitive biases that shape linguistic systems may be relatively strong in initial stages of learning, weak biases that persist in development can still exert pressure for language change over generations (Kirby et al., 2008; Thompson, Kirby, & Smith, 2016). The extent to which these pressures in natural languages come from child learners or adults remains less clear and may be specific to the phenomenon in question. For example, nonharmonic languages may be more susceptible to change through influence from language contact, sociolinguistic variation, or the introduction of second language learners to the population (Labov, 1994; Trudgill, 2001). These are all sources of variation, which may trigger observable effects from the weak biases of adult language users. Alternatively, word order change may be driven by small deviations from the input due to reanalysis in language acquisition by children. Further research is needed to better understand the potential role of these precise mechanisms in linking the harmony bias to language typology.

## ACKNOWLEDGMENTS

Thanks to Sarah Furlong and to participants and their parents. This research was partially supported by NIH Grants HD37082 and DC014558 and by funds from the Center for Brain Plasticity and Recovery, Georgetown University.

## AUTHOR CONTRIBUTIONS

JC and ELN designed the research, ELN supervised data collection, JC analyzed data, JC and ELN wrote the article.

## Notes

^1^ In the 6- to 7-year-old group, 11 additional children were tested but not included in the analysis due to technical problems (2), failure to complete the training (1), and failure to complete more than two critical trials for each modifier type (6). In the 4- to 5-year-old group, 27 additional children were tested but not included in the analysis due to technical problems (3), failure to complete the training (14), and failure to complete more than two critical trials for each modifier type (7). Training in this experiment was somewhat lengthy due to the need for participants to learn to produce the nonce words, and many of the youngest children elected to stop. The exclusion based on number of trials was motivated by the need for sufficient responses for each modifier in order to determine a word order preference for each child. This left an average of 43 (*SD* = 7) and 31 (*SD* = 15) trials in the 6- to 7- and 4- to 5-year-old groups, respectively. There is no indication of any difference in results in the partial data for those who did not complete the experiment.^2^ Random slopes for noun and modifier (i.e., particular adjective or numeral) are included when comparing among child groups. These are not included when comparing adults and children as the lexicon differed across them. In the main text we report significance on the basis of likelihood-ratio tests. Main effects in the presence of interactions were calculated by converting the factor not being tested into a sum-coded numeric representation and conducting a likelihood-ratio test between models differing only in whether they included a fixed main effect of the factor of interest (see Levy, 2014, for discussion of this approach).^3^ All significant and nonsignificant effects remain so when *p* values are calculated used the Wald *z* statis tic instead (all factors sum coded): effect of condition on input order use (β = −0.26 ± 0.28, *p* = .36), effect of age (adult vs. child) (β = 3.26 ± 0.47, *p* < .001), effect of age (4–5yrs vs. 6–7yrs) (β = 0.20 ± 0.17, *p* = .23), effect of vocabulary accuracy in children (β = −0.10 ± 0.06, *p* = .11), effect of condition in children (β = −0.19 ± 0.17, *p* = .26), and interaction between vocabulary accuracy and age in children (β = −0.13 ± 0.60, *p* = .03).^4^ All effects reported as significant remain so if *p* values are calculated based on Wald *z* score (all factors sum coded): effect of age (4–5yrs vs. 6–7yrs) on choice of harmonic pattern (β = −0.75 ± 0.37, *p* = .03); effect of pattern type (harmonic vs. non-harmonic) on proportion use of preferred pattern (β = −0.06 ± 0.03, *p* = .10); effect of harmonic pattern (pre- or postnominal) on proportion use of preferred pattern (β = −0.04 ± 0.03, *p* = .21).
